# Patient-reported outcome measures in patients with familial cerebral cavernous malformations: results from the Treat_CCM trial

**DOI:** 10.3389/fneur.2024.1338941

**Published:** 2024-02-14

**Authors:** Jennifer M. T. A. Meessen, Giorgia Abete-Fornara, Barbara Zarino, Marco Castori, Laura Tassi, Maria R. Carriero, Q. G. D'Alessandris, R. Al-Shahi Salman, Adriana Blanda, Enrico B. Nicolis, Deborah Novelli, Maria Caruana, Antonella Vasamì, Silvia Lanfranconi, Roberto Latini

**Affiliations:** ^1^Department of Acute Brain and Cardiovascular Injury, Institute for Pharmacological Research Mario Negri IRCCS, Milan, Italy; ^2^Department of Neurochirurgia, Fondazione IRCCS Cà Granda Ospedale Maggiore Policlinico, Milan, Italy; ^3^Division of Medical Genetics, Fondazione IRCCS Casa Sollievo della Sofferenza, San Giovanni Rotondo, Italy; ^4^"Claudio Munari" Epilepsy Surgery Centre, ASST Grande Ospedale Metropolitano Niguarda, Milan, Italy; ^5^Cerebrovascular Disease Unit, Fondazione IRCCS Istituto Neurologico Carlo Besta, Milan, Italy; ^6^Department of Neurosurgery, Fondazione Policlinico Universitario Agostino Gemelli IRCCS, Università Cattolica del Sacro Cuore, Rome, Italy; ^7^Centre for Clinical Brain Sciences, University of Edinburgh, Edinburgh, United Kingdom; ^8^Department of Neurology, Fondazione IRCCS Cà Granda Ospedale Maggiore Policlinico, Milan, Italy

**Keywords:** familial cerebral cavernous malformation, neurology, patient-reported outcome measures (PROMs), depression, anxiety, quality of life

## Abstract

**Background:**

The Phase 1/2 Treat_CCM randomized controlled trial for people with familial cerebral cavernous malformations (FCCMs) confirmed the safety of propranolol and suggested beneficial effects on intracerebral hemorrhage or new focal neurological deficits, but the effects on patient-reported outcome measures have not been reported.

**Methods:**

Participants completed self-reported questionnaires at baseline, 1 and 2 years. Depression was assessed with the Beck Depression Inventory-II (BDI-2); Anxiety with the State–Trait Anxiety Inventory X1 and X2 (STAI X-1 and STAI X-2); and Quality of Life with the Short Form 36 (SF-36), split into the physical and mental component scales (PCS and MCS). Differences between treatment groups and the general population were assessed. Change over time by treatment was assessed by means of mixed models.

**Results:**

In total, 71 participants (48 propranolol and 23 standard care) were enrolled, of whom 61 (73%) completed questionnaires at baseline and 2-year FU. At baseline, no differences between treatment groups for any of the questionnaires were present. Twenty (31.7%) patients were considered depressed at baseline, while this proportion was lower in the propranolol group after 2 years (28.6% vs. 55.5%, *p* = 0.047). The STAI X-1 and X-2 scores were stable over time. PCS was lower in FCCM patients as compared with the general Italian population, while the MCS was similar to the general population. No effect of propranolol was found for both PCS and MCS.

**Conclusion:**

Depression is common among patients with FCCM. Patients randomized to propranolol had a lower proportion of participants with depression after 2 years.

**Clinical trial registration**: https://clinicaltrials.gov/, identifier (NCT03589014).

## Background

Familial cerebral cavernous malformation (FCCM) is a rare subtype of CCM with a prevalence of 0.1–0.8% (1), which is inherited as an autosomal dominant trait and is characterized by multiple cavernomas. Cerebral cavernomas progressively increase in number and dimensions during a patient’s life. Leakage and instability of such lesions cause focal neurological deficits (FNDs), seizures, and recurrent intracerebral hemorrhages (ICHs), which highly affect the quality of life (QoL) and life expectancy of affected individuals. Two studies by two independent groups in different murine models of CCM prompted an assessment of propranolol effects in a controlled clinical trial. The proposed mechanisms of action of propranolol are different, while both studies agree on the beneficial effects of the drug. Li et al. (2) suggest that the benefit of propranolol is mediated by the blockade of beta-1 receptors, while Oldenburg et al. (3) show that propranolol has the capacity to not only reduce the lesion burden but also improve vascular stability through a protective action on pericytes.

However, in the literature, many studies can be found concerning the surgical outcome or neurological characteristics of patients with this disease; studies assessing QoL and mental wellbeing in cerebral or brainstem cavernomas are very rare, and, to our knowledge, no studies have been conducted specifically on FCCM patients so far.

Although patients with cerebral CCM lesions have been reported to show better levels of QoL compared to patients with brainstem lesions (4, 5), previous reports describe significant physical limitations and decreased mental health in CCM patients. For example, Herten et al. (6) assessed QoL in a non-invasively treated group of CCM patients, including some cases of FCCM, reporting a significant decrease in QoL levels and worse mental health even in the absence of functional or neurological impairments. Decreases in QoL, loss of autonomy in daily life activities, and self-care and/or mental health problems were found in approximately 30–40% of CCM patients, including the hereditary FCCMs (7–9). In these studies, using different self-report questionnaires, patients frequently reported problems concerning physical limitations, inability to work, anxiety toward the future, and general health and depression, showing how relevant QoL and mental health measures are to understand the real impact of a medical condition on a patient’s daily life. Opposite results have been described: Cornelius and colleagues report no differences in physical and mental health between CCM patients and the normative healthy population (4). Thus, controversial results seem to emerge from the few studies available in the literature, underlying the need for further research to better understand the actual impact of CCM on patients’ daily lives.

This study presents QoL and mental health data concerning a large group of patients with FCCM participating in a pharmacological trial, the Treat_CCM trial, which assessed the effect of propranolol on the disease course (10). The aim of the present study was to assess the QoL, anxiety, and depression data of FCCM patients over 2 years and to verify whether propranolol has an effect on these variables.

## Methods

### Study design

Treat_CCM was a phase 1/2, randomized, open-label, blinded endpoint pilot trial conducted in six Italian hospitals: Fondazione IRCCS Ca’ Grande Ospedale Maggiore Policlinico (Milano), Fondazione Policlinico Universitario. A. Gemelli (Roma), Fondazione IRCCS Istituto Neurologico Carlo Besta (Milano), Fondazione IRCCS Casa Sollievo della Sofferenza (San Giovanni Rotondo), ASST Grande Ospedale Metropolitano Niguarda, Milan, and IRCCS Centro Neurolesi “Bonino Pulejo” (Messina). Seventy-one patients with symptomatic FCCM aged ≥18 years were included.

Patients were randomly assigned 2:1 to propranolol (10–160 mg/12 h) with standard care or standard care alone for 24 months. Investigators performed clinical evaluations and 3-Tesla brain magnetic resonance imaging (MRI) at baseline, 12 and 24 months. The primary outcome was a new occurrence of symptomatic intracerebral hemorrhage (ICH) or focal neurological deficit (FND) attributable to CCM. Outcome assessors—but not participants, caregivers, or investigators—were blinded to group assignment. Treat_CCM is registered with EudraCT (2017–003595-30) and ClinicalTrials.gov (NCT03589014). Protocol details and trial results have been previously published (10, 11). All local research ethics committees approved the study, which has been performed in accordance with the ethical standards laid down in the 1964 Declaration of Helsinki and its later amendments. All participants provided written informed consent at the first visit before any study procedures or assessments. The trial adhered to good clinical practice (GCP) requirements and all the applicable regulatory requirements. The trial was completed in December 2021.

### Mental health and quality of life assessments

Clinical outcomes, other than ICH and FND, such as health-related quality of life (QoL) and mental health (intended as levels of anxiety and depression), were assessed by means of patient-reported outcome measures (PROMs). Patients with familial CCM who participated in the Treat_CCM trial were asked to complete PROMs at baseline, after 1 year, and at the end of the trial (2 years). Most of the patients completed the questionnaires during one of the face-to-face clinical appointments with a clinician, a psychologist (where available), or a neurologist. In some cases, if the patient was unable to reach the hospital or to complete the questionnaires during the scheduled visit, questionnaires were sent by e-mail after a deep and precise explanation of how to respond. When questionnaires were sent back, a clinician carefully examined if they were completely and correctly filled in.

- Depression was assessed by means of the Beck Depression Inventory—II (BDI-II). This scale allows us to distinguish the following levels of depression: score 0–9 no depression; score 10–18 slight depression; score 19–29 moderate depression; score 30–63 severe depression. Patients were classified as depressed with a cutoff of 10 (12).
- Anxiety was assessed by means of the Short Form Health Survey (forms X-1 and X-2, STAI X-1 and STAI X-2) (13). The STAI X-1 measures the level of anxiety at the moment of completing the questionnaire (state anxiety), while the STAI X-2 assesses the level of anxiety a person tends to experience in general; thus, it is a measure of a personality characteristic (trait anxiety). A patient is classified as being in a state of anxiety while completing the questionnaire if (s)he scores in the top 99% (for male individuals, this corresponds to a score of ≥65, and for female individuals, it is ≥71), while for trait anxiety, this is the top 95% (for male individuals, this corresponds to a score of ≥56, and for female individuals, it is ≥62).
- QoL was assessed by means of the SF-36. The SF-36 comprises eight subdomains: physical functioning (PF), social functioning (SF), role limitations due to physical problems (RPs), role limitations due to emotional problems (RE), general mental health (MH), vitality (VT), bodily pain (BP), and general health (GH) perceptions. Each subdomain has a score from 0 (worst) to 100 (best). These subdomains can be combined into the physical component score (PCS) and the mental component score (MCS). A higher score indicates a better QoL (14, 15).

### Statistical analysis

Baseline characteristics are presented as mean ± standard deviation (SD), median [Q1–Q3], or N (%), as appropriate, based on their distribution as assessed by means of the Shapiro–Wilk test. Differences in patient characteristics between those with and without depression were assessed by means of Kruskal–Wallis or Chi^2^-tests. The variables (excluding treatment for depression) that showed differences between depressed and non-depressed patients with *p* < 0.200 were included in linear regression analysis to assess which variables were independently associated with the BDI-II score. Change over time for continuous BDI-II score within patients between treatment groups was assessed by means of a mixed-model ANOVA. Patient characteristics between persons with and without anxiety were assessed by means of Kruskal–Wallis or Chi^2^-tests. Scores on the SF-36 subdomains of the Treat_CCM population were compared to the scores of the general Italian population as described in Apolone and Mosconi (14). Patients were split into groups according to median MCS and PCS scores and compared by means of Kruskal–Wallis or Chi^2^ tests. The effect of treatment on the continuous MCS score over time was assessed by means of mixed-model ANOVA. *p* < 0.05 after correction for multiple testing by fdr-correction were considered statistically significant.

## Results

### Patient demographics

The 71 patients with FCCM included in the Treat_CCM trial were generally without significant comorbidities. The average age was 46 years, and 56% were women. A total of 65% of the patients had experienced an ICH in their lifetime and 48% had experienced FND. A total of 42.3% of the patients suffered from CCM-related epilepsy. Sixteen (22.5%) patients had C-reactive protein (CRP) levels above 2 mg/L, an indication of inflammation. Nine (12.7%) patients were on antidepressants at baseline.

Sixty-one patients completed all questionnaires at baseline and after 2 years. Patients who did not complete the questionnaires experienced ICH more often during the course of the study (p_fdr_ = 0.016) (Supplementary Table S1).

### Depression

BDI at baseline was available for 63 people. At baseline, the median score for BDI-II was 6.0 [4.0–11.0], with a maximum score of 26. A score of 10 indicates a clinically significant level of depression and was present in 20 (31.7%) patients. Patients depressed at baseline were significantly older than non-depressed patients (age 53 ± 15 years vs. 43 ± 14 years, p_fdr_ = 0.049). Depressed patients had a non-significantly higher lifetime frequency of CCM-related events such as ICH (70.0% vs. 58.1%, p_fdr_ = 0.587) and FND (65.0% vs. 39.5%, p_fdr_ = 0.203) and had a higher score on the Modified Rankin Scale (mRS) for disability (1 [1–3] vs. 0[0–1], p_fdr_ = 0.019 respectively). However, seizure history did not affect depression (40.0% in depressed patients vs. 37.2% in non-depressed patients, p_fdr_ = 0.851). Antiseizure treatment was slightly elevated in depressed patients (55.0% vs. 39.5%, p_fdr_ = 0.587). Only 7 of the 20 depressed patients were on antidepressant treatment, while 2 out of 43 non-depressed patients were on antidepressants. There were marked differences between depressed and non-depressed patients for all other PROMs at baseline, with the depressed patients having significantly worse scores (pfdr < 0.05) (see Supplementary Table S2). Linear regression analysis including age, time since diagnosis, history of FND, cholesterol level, triglycerides, CRP, mRS score, and antihypertensive treatment showed that triglycerides (B = 0.039 [95%CI: 0.007–0.072]) and mRS (B = 1.174 [95%CI: 0.092–2.256]) were independently associated with an increased score on the BDI scale at baseline. CRP, a marker of inflammation, was increased at baseline in depressed patients as compared to non-depressed patients (1.75 [0.76–4.44] mg/L vs. 0.70 [0.31–1.30] mg/L).

**Figure 1:**
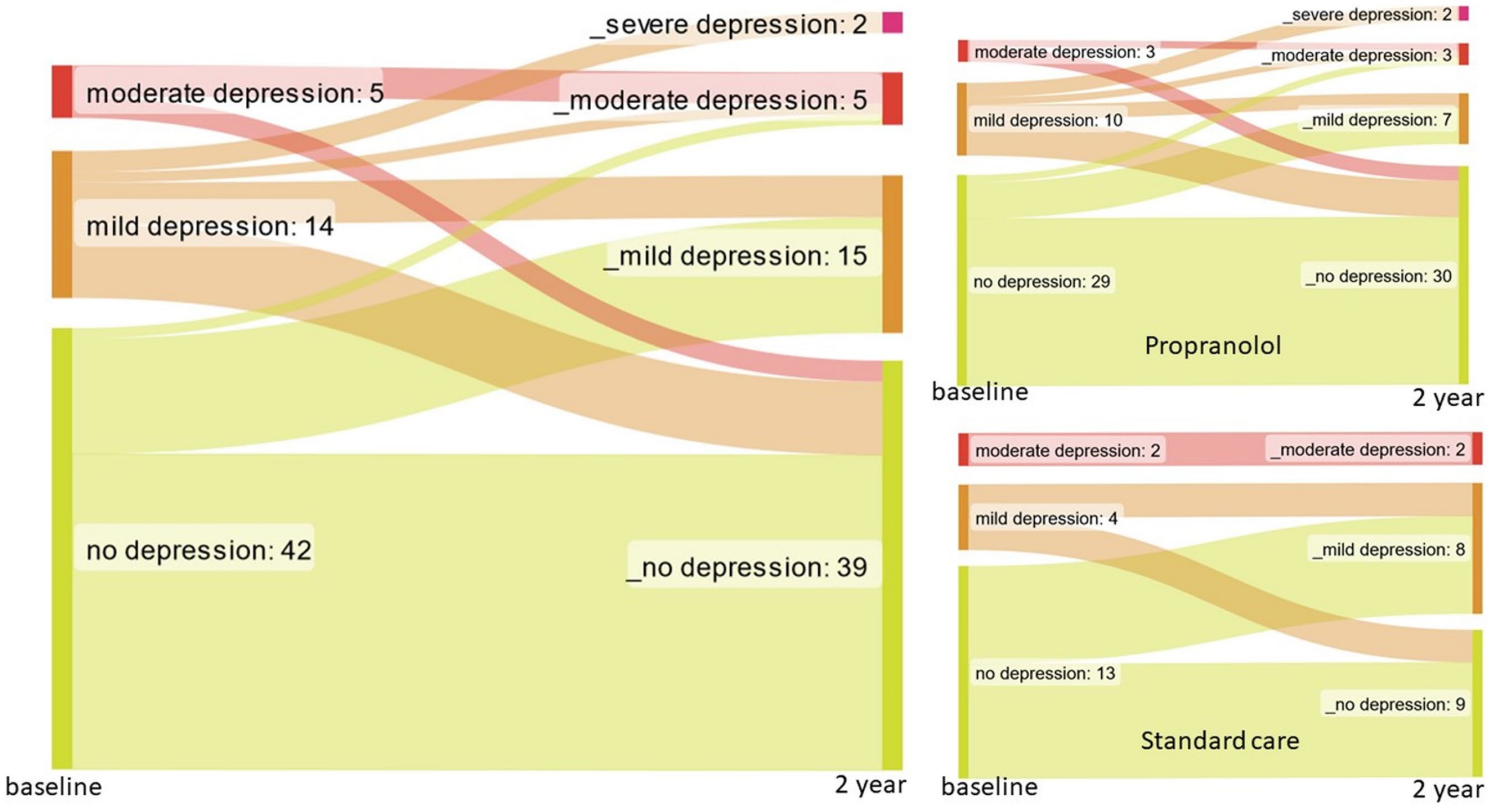
Flow of patients for depression at baseline and 2 years follow-up.

Sixty persons had BDI available at both baseline and 2 years. The BDI score slightly increased over 2 years (median score at 2-year FU 7.5 [3.0–11.0], maximum score 37) as compared to baseline (6.0 [4.0–11.0], maximum score 26, *p* = 0.377), suggesting worse depressive symptoms. There was no significant difference between the propranolol-treated group and the control group with respect to the 2-year change in BDI score (*p* = 0.659). In Figure 1, the flow of patients during the 2 years of follow-up is depicted. At baseline, the proportions of depressed patients were similar between treatment groups [propranolol 13 (31.0%) vs. standard care 6 (33.3%) patients]. After 2 years, there was a difference in the proportion of depressed patients: 12 (28.6%) in propranolol and 10 (55.5%) in standard care (*p* = 0.047).

### Anxiety scores

Two types of anxiety were measured: current state anxiety (STAI X-1) and trait anxiety (STAI X-2). The STAI X-1 and STAI X-2 median scores were 40 [32–49] and 41 [33–47] at baseline, respectively. These scores were stable during the course of the trial, with 39 [33–53] and 41 [33–48] after a 2-year follow-up. At baseline, none of the patients were considered to be in a current state of anxiety; however, at the end of the study, one patient was considered anxious. STAI X-2 scores ranged from 20 to 77, with higher scores indicating a worse level of trait anxiety. The STAI X-2 scores were stable over time: the median score at baseline was 41 [33–47] and at 2 years, 41 [33–48]. A patient was considered to experience significant trait anxiety if male individuals scored ≥56 and female individuals ≥62 on the STAI X-2 questionnaire. At baseline, three patients were considered to show trait anxiety; two of these also experienced state anxiety at 2 years, and the other trait-anxious patient had his/her questionnaire missing. In addition, one patient became generally anxious (STAI X-2 clinically significant) during the study. Supplementary Table S3 depicts the differences between non-anxious patients and those who, at any moment during the trial, scored as anxious at either STAI X-1 or STAI X-2. Anxious patients were more often treated with antidepressant drugs (50.0% vs. 9.2%, p_fdr_ = 0.029) and scored significantly worse on all scales at baseline. Although there was no difference in baseline anxiety for patients with or without a history of ICH or FND (p_fdr_ = 0.950 for both), there was a higher proportion of ICH events during follow-up in the anxious patient group (16.7% vs. 1.5%, p_fdr_ = 0.141). Seizure history did not affect anxiety.

### QoL

The radar plots in Supplementary Figure S1 show the score on the eight individual subdomains of the SF-36 questionnaire for the general Italian population (green) and the Treat_CCM treatment groups after 2 years of treatment. A higher score indicates increased QoL. In the SF-36, only one domain, “Role Physical,” showed a clear difference between the propranolol-treated, control, and Italian population (68.1 ± 39.9 vs. 51.1 ± 43.3 vs. 78.2 ± 35.9, respectively); however, this did not reach statistical significance. PF, SF, RE, VT, BP, and GH showed a clinically significant decrease of 2 points, but these results did not reach statistical significance. No differences were found for MH.

The subdomains were regrouped into the mental component score (MCS) or physical component score (PCS). At baseline, the PCS of the Treat_CCM population was below the general population: 44.9 ± 10.3, while the general Italian population scored 52.7 ± 7.7. This difference was higher than the minimally clinically important difference of two points (12). For the MCS, there were no differences: Treat_CCM patients scored on average 47.7 ± 15.1 at baseline, while the general population scored 47.6 ± 10.1.

Upon splitting the patients by MCS score of 50, patients with lower MCS (meaning lower QoL) had increased levels of CRP and creatinine. Patients with low MCS had significantly higher scores on the BDI and STAI questionnaires, indicating increased depression and anxiety. No statistically significant differences were found for PCS at baseline; however, the median for both groups differed by 2 points, a clinically significant difference (MCS < 50: 46.1 [38.1–51.5]; MCS ≥50: 48.8 [36.6–52.1], p_fdr_ = 0.896) (see Supplementary Table S4). Patients with a higher PCS score had a lower proportion of FND in life as compared to those with a lower PCS (25.0% vs. 62.8%, p_fdr_ = 0.015). In addition, patients with an increased score on the PCS had lower scores on the BDI and STAI questionnaires, indicating less depression and anxiety. Although no effect of seizures during the course of the study was found for depression, anxiety, or MCS, the three patients who did experience an epileptic seizure during the course of the Treat_CCM trial scored all three ≥50 for PCS (p_fdr_ = 0.123). A large, non-statistically significant difference was found for MCS (PCS < 50: 44.2 [28.5–61.0]; PCS ≥50: 58.6 [43.4–61.0], p_fdr_ = 0.281) (see Supplementary Table S5).

**Figure 2:**
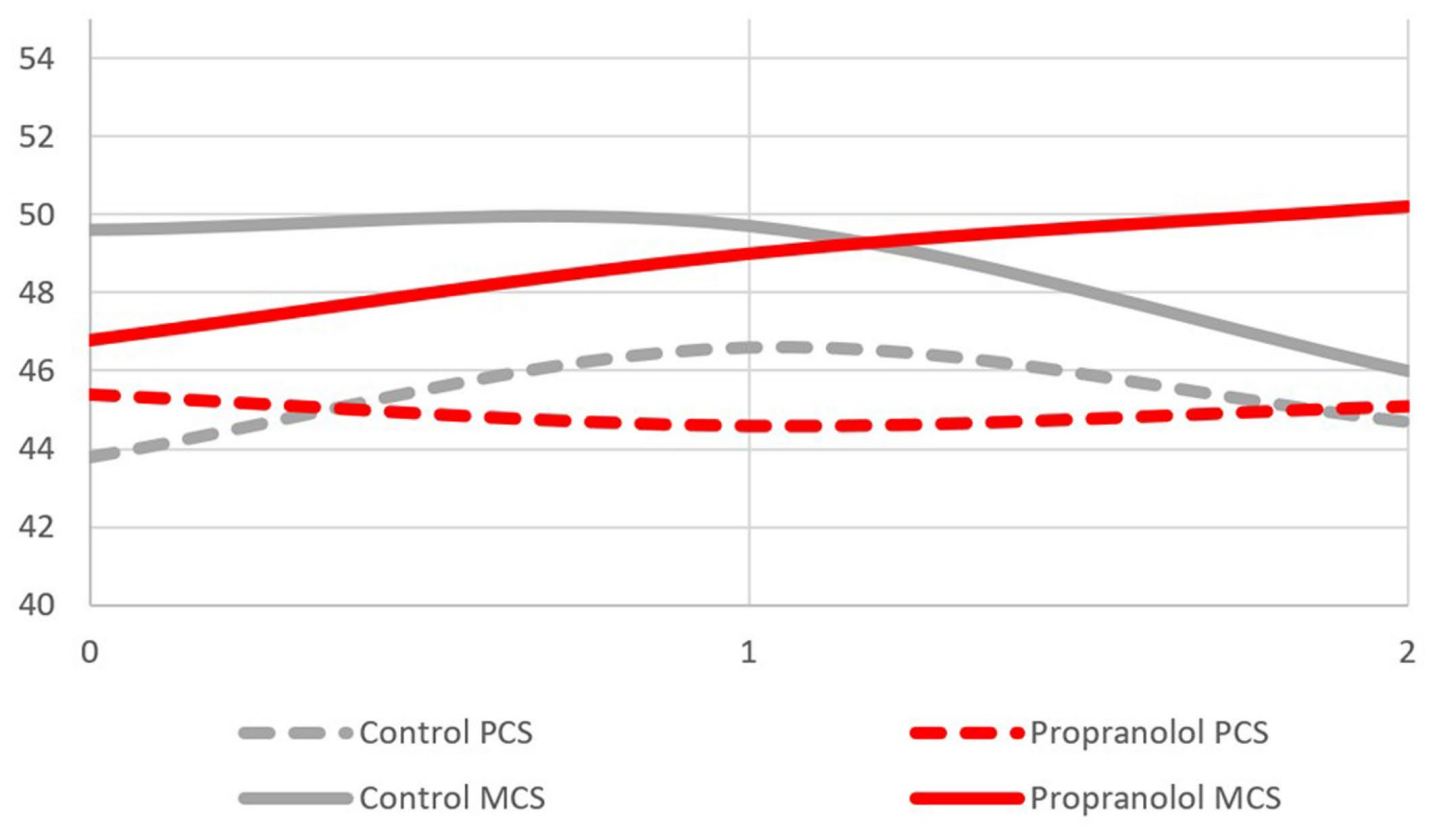
PCS and MCS score over time by treatment group.

While no difference was observed for the PCS score after 2 years of follow-up (propranolol 45.1 ± 9.3; standard care 44.7 ± 10.5), patients randomized to propranolol had an increased MCS as compared to standard care (50.2 ± 15.1; standard care 46.0 ± 15.4, *p* = 0.198). Mixed model analysis for the effect of propranolol on the development of the continuous MCS score over time showed a borderline significant interaction of treatment and time (*p* = 0.065) (see Figure 2).

## Discussion

This study presents prospective data collected over 2 years from a series of PROMs investigating QoL and mental health in a group of 71 patients with FCCM enrolled in Treat_CCM, the first randomized controlled clinical trial comparing the safety and efficacy of propranolol. In general, we reported a positive or neutral effect of propranolol on daily life autonomy and psychological wellbeing, suggesting that the drug was well tolerated.

Considering psychological health, almost one-third of the patients suffered from depression (defined as BDI score ≥ 10) at baseline; this substantial proportion was associated with older age, a more severe clinical condition and disability, and a higher plasma concentration of an inflammatory circulating biomarker, CRP. Interestingly, these data are in line with a previous finding by Herten et al., describing increased levels of depression (measured with another clinical PROM than ours) in a group of untreated patients with sporadic and familial CCM, although the percentage of depressed patients was comparable to the general German population (6). Other recent studies found no differences between CCM patients and the healthy population in depression levels (8, 9). In our study, no significant changes over time in depression levels were found, although after 2 years of propranolol, patients tended to be less depressed than those in the standard care group. This element may suggest a positive role of propranolol in the general clinical condition of patients, leading to better mental wellbeing.

As far as anxiety is concerned, no significant levels of state or trait anxiety were found at any time point, indicating a stable anxiety level over time with no differences between the two treatment groups. The intake of propranolol did not increase anxiety levels. Propranolol was used in the past decades to treat anxiety symptoms before the massive use of benzodiazepines; no significant changes in anxiety levels emerged in our sample, considering that at baseline no significant state or trait anxiety levels were present, thus we may exclude a significant interference of propranolol on anxiety symptoms (16). Moreover, a recent review and meta-analysis proved no significant efficacy of this drug on a variety of anxiety disorders.

Previous reports described increased anxiety in patients with CCM (6, 8, 9), a further element that suggests a neutral role of propranolol over clinical anxiety in Treat_CCM. A recent study found a significant effect of gender on anxiety levels, with the female population suffering the most. Although not significant, the number of women suffering from anxiety was elevated in our sample (8). Anxiety levels were significantly associated with older age, the presence of spinal CCM, or more severe depressive symptoms.

Even if it could be expected that propranolol might cause physical discomfort by lowering blood pressure and heart rate, patients randomized to propranolol did not perceive a significant decrease in their physical autonomy, and their physical health was not affected by the assumption of propranolol. In contrast, MCS was increased in the propranolol group as compared with patients randomized to standard care after 2 years of treatment, thus suggesting a protective role of propranolol over psychological wellbeing. However, a subjective bias cannot be excluded given the open design of Treat_CCM.

The follow-up of Treat_CCM was 2 years, and clinical events such as ICH or FND were consistent with the expected events (17). The 2-year follow-up allowed for only a few cases with clinical events in the study, which in turn also made them less likely to complete the questionnaires during the course of the study. Therefore, a longer follow-up would have allowed for more data on PROMs and probably a more pronounced difference.

The IQ levels of the participating patients were not collected in the Treat_CCM study as they did not impact the questionnaires used, while other demographic data such as age or gender were, as reported in the respective manuals and normative data. Thus, the effect of the patient’s IQ on the PROMs could not be assessed.

In the end, it is important to consider the particular management our patients received; in fact, several clinicians (such as the neurologist, the radiologist, and the case manager of the center) closely followed these patients through the whole duration of the trial. This multidisciplinary management is one of the features that allows the patient to feel taken into consideration as a person, an important element also in terms of compliance related to mood and QoL. A placebo effect cannot be excluded given the open design of the trial; nevertheless, patients are not experienced in propranolol’s possible clinical effects on psychological health, and, as the recent pandemic demonstrated, new drugs can also cause opposite effects than the placebo due to patients’ fear of taking a new treatment not already approved for their pathology.

The literature reports significantly decreased levels of QoL in CCM patients as compared to the normal population, with the former group showing decreased scores in both the domains of mental and physical functioning (8, 9).

## Conclusion

In conclusion, in addition to being safe and well tolerated, as recently reported (10), propranolol seems to have a neutral effect on depression and anxiety and may have a mild positive influence on certain domains of the SF-36. Future research could further analyze the relationship between propranolol, QoL, and psychological health, and other clinical factors, such as the brain areas involved, a parameter considered to be of clinical interest (18).

## Data availability statement

The dataset generated in this study is available in the Zenodo repository (https://zenodo.org/records/10497617).

## Ethics statement

The studies involving humans were approved by local ethics boards of each participating hospital. The studies were conducted in accordance with the local legislation and institutional requirements. The participants provided their written informed consent to participate in this study.

## Author contributions

JM: Formal analysis, Investigation, Methodology, Visualization, Writing – original draft. GA-F: Formal analysis, Investigation, Methodology, Visualization, Writing – original draft. BZ: Conceptualization, Methodology, Writing – review & editing. MCas: Data curation, Writing – review & editing. LT: Data curation, Writing – review & editing. MRC: Data curation, Writing – review & editing. QD'A: Data curation, Writing – review & editing. RA-S: Investigation, Methodology, Supervision, Writing – review & editing. AB: Formal analysis, Writing – review & editing. EN: Data curation, Software, Writing – review & editing. DN: Investigation, Writing – review & editing. MCar: Data curation, Project administration, Writing – review & editing. AV: Data curation, Project administration, Writing – review & editing. SL: Conceptualization, Data curation, Funding acquisition, Investigation, Resources, Supervision, Writing – review & editing. RL: Conceptualization, Funding acquisition, Investigation, Methodology, Resources, Supervision, Writing – review & editing.

## Glossary

BDI-2: Beck Depression Inventory-II
BP: Bodily pain
CCM: Cerebral cavernous malformations
CRP: C-reactive protein
fCCM: Familial cerebral cavernous malformations
FND: Focal neurological deficits
GCP: Good clinical practice
GH: General health perceptions
ICH: Intracerebral hemorrhages
MCS: Mental component scale
MH: General mental health
mRS: Modified Rankin Scale
PCS: Physical component scale
PF: Physical functioning
PROMs: Patient-reported outcome measures
QoL: Quality of life
RE: Role limitations due to emotional problems
RP: Role limitations due to physical problems
SD: Standard deviation
SF: Social functioning
SF-36: Short Form 36
STAI X-1: State–Trait Anxiety Inventory X-1
STAI X-2: State–Trait Anxiety Inventory X-2
VT: Vitality

## References

[1] Zafar A Quadri SA Farooqui M Ikram A Robinson M Hart BL. Familial cerebral cavernous malformations. Stroke. (2019) 50:1294–301. doi: 10.1161/STROKEAHA.118.022314 30909834 PMC6924279

[2] Li W Shenkar R Detter MR Moore T Benavides C Lightle R. Propranolol inhibits cavernous vascular malformations by β1 adrenergic receptor antagonism in animal models. J Clin Invest. (2021) 131:e154909. doi: 10.1172/JCI154909, PMID: 34596055 PMC8483741

[3] Oldenburg J Malinverno M Globisch MA Maderna C Corada M Orsenigo F. Propranolol reduces the development of lesions and rescues barrier function in cerebral cavernous malformations: a preclinical study. Stroke. (2021) 52:1418–27. doi: 10.1161/STROKEAHA.120.029676 33618555

[4] Cornelius JF Kürten K Fischer I Hänggi D Steiger HJ. Quality of life after surgery for cerebral Cavernoma: brainstem versus nonbrainstem location. World Neurosurg. (2016) 95:315–21. doi: 10.1016/j.wneu.2016.08.014 27542564

[5] Shoubash L Baldauf J Matthes M Kirsch M Rath M Felbor U. Long-term outcome and quality of life after CNS cavernoma resection: eloquent vs. non-eloquent areas. Neurosurg Rev. (2022) 45:649–60. doi: 10.1007/s10143-021-01572-8 34164745 PMC8827309

[6] Herten A Chen B Saban D Santos A Wrede K Jabbarli R. Health-related quality of life in patients with untreated cavernous malformations of the central nervous system. Eur J Neurol. (2021) 28:491–9. doi: 10.1111/ene.14546, PMID: 32961598

[7] Bicalho VC Bergmann A Domingues F Frossard JT de Souza JPBM. Cerebral cavernous malformations: patient-reported outcome validates conservative management. Cerebrovasc Dis. (2017) 44:313–9. doi: 10.1159/000480125, PMID: 28968597

[8] Kim H Flemming KD Nelson JA Lui A Majersik JJ Cruz MD. Baseline characteristics of patients with cavernous Angiomas with symptomatic hemorrhage in multisite trial readiness project. Stroke. (2021) 52:3829–38. doi: 10.1161/STROKEAHA.120.033487, PMID: 34525838 PMC8608704

[9] Rauschenbach L Bartsch P Santos AN Lenkeit A Darkwah Oppong M Wrede KH. Quality of life and mood assessment in conservatively treated cavernous malformation-related epilepsy. Brain Behav. (2022) 12:e2595. doi: 10.1002/brb3.2595, PMID: 35470577 PMC9226805

[10] Lanfranconi S. Safety and efficacy of propranolol for treatment of familial cerebral cavernous malformations (Treat_CCM): a randomised, open-label, blinded-endpoint, phase 2 pilot trial. Lancet Neurol. (2023) 22:35–44. doi: 10.1016/S1474-4422(22)00409-4 36403580

[11] Lanfranconi S Scola E Bertani GA Zarino B Pallini R d’Alessandris G. Propranolol for familial cerebral cavernous malformation (Treat_CCM): study protocol for a randomized controlled pilot trial. Trials. (2020) 21:401. doi: 10.1186/s13063-020-4202-x, PMID: 32398113 PMC7218540

[12] Salkind MR. Beck depression inventory in general practice. J R Coll Gen Pract. (1969) 18:267–71. PMID: 5350525 PMC2237076

[13] Spielberger CD Sydeman SJ Owen AE Marsh BJ. Measuring anxiety and anger with the state-trait anxiety inventory (STAI) and the state-trait anger expression inventory (STAXI) In: The use of psychological testing for treatment planning and outcomes assessment. 2nd ed. Mahwah, NJ: Lawrence Erlbaum Associates Publishers (1999). 993–1021. Avaialable at: https://psycnet.apa.org/record/1999-02767-031

[14] Apolone G Mosconi P. The Italian SF-36 health survey: translation, validation and norming. J Clin Epidemiol. (1998) 51:1025–36. doi: 10.1016/s0895-4356(98)00094-8 9817120

[15] Ware JE. SF-36 health survey update. Spine. (2000) 25:3130–9. doi: 10.1097/00007632-200012150-00008 11124729

[16] Steenen SA van Wijk AJ van der Heijden GJMG van Westrhenen R de Lange J de Jongh A. Propranolol for the treatment of anxiety disorders: systematic review and meta-analysis. J Psychopharmacol. (2016) 30:128–39. doi: 10.1177/0269881115612236 26487439 PMC4724794

[17] Horne MA Flemming KD Su I-C Stapf C Jeon JP Li D. Clinical course of untreated cerebral cavernous malformations: a meta-analysis of individual patient data. Lancet Neurol. (2016) 15:166–73. doi: 10.1016/S1474-4422(15)00303-8 26654287 PMC4710581

[18] Akers A al-Shahi Salman R Awad IA Dahlem K Flemming K Hart B. Synopsis of guidelines for the clinical management of cerebral cavernous malformations: consensus recommendations based on systematic literature review by the Angioma Alliance scientific advisory board clinical experts panel. Neurosurgery. (2017) 80:665–80. doi: 10.1093/neuros/nyx091, PMID: 28387823 PMC5808153

