# Supplementary material for: Patient-reported outcome measures in patients with familial cerebral cavernous malformations: results from the Treat_CCM trial
Source: Front Neurol. 2024 Feb 14;15:1338941. doi: 10.3389/fneur.2024.1338941 (PMC10899450; doi:10.3389/fneur.2024.1338941)
Supplement: Supplementary file 1 [file Data_Sheet_1.PDF]

Supplementary material for
Patient reported outcome measures of
FCCM patients in Treat_CCM

Supplementary Table 1 – Baseline characteristics for those who completed and did not complete the questionnaires**.**

Supplementary Table 2 – Baseline characteristics of patients with FCCM who were depressed at baseline**.**

Supplementary Table 3 – Baseline characteristics of patients with FCCM who were anxious at baseline**.**

Supplementary Table 4 – Baseline characteristics of patients with FCCM with respect to Mental Component Scale (MCS) at baseline**.**

Supplementary Table 5 – Baseline characteristics of patients with FCCM with respect to Physical Component Scale (PCS) at baseline**.**

Supplementary Figure 1 – SF domains for Treat_CCM groups and the Italian general population.

Supplementary Table 1 – Baseline characteristics for those who completed and did not complete the questionnaires.

		Familial CCM (N=71)	Incomplete (N=10)	Complete (N=61)	P	P_{fdr}
**Age** (years)	Baseline	45.5 ± 14.9	48.4 ± 18.6	45.0 ± 14.3	0.457	0,836
**Sex** (female)	Baseline	40 (56.3%)	5 (50.0%)	35 (57.4%)	0.663	0,861
**BMI** (kg/m^2)	Baseline	24.0 ± 3.6	22.6 ± 3.3	24.3 ± 3.6	0.211	0,666
**Randomized to propranolol**	Baseline	48 (67.6%)	6 (60.0%)	42 (68.9%)	0.579	0,836
**Mean dose**	Baseline	73.6 ± 44.3	66.7 ± 45.9	74.6 ± 44.5	0.999	0,999
**Systolic BP** (mmHg)	Baseline	122 ± 14	123.5 ± 14.5	121.4 ± 14.1	0.874	0,933
**Diastolic BP** (mmHg)	Baseline	79 ± 8	76.5 ± 4.7	78.9 ± 8.7	0.262	0,675
**Heart rate** (bpm)	Baseline	71 ± 12	70.9 ± 14.6	71.5 ± 11.5	0.797	0,914
**Genetics**	KRIT1	54 (76.1%)	8 (80.0%)	46 (75.4%)	0.635	0,861
	MGC4607	12 (16.9%)	2 (20.0%)	10 (16.4%)		
	PDCD10	5 (7.0%)	0	5 (8.2%)		
**Time since diagnosis**	Baseline	16.1 ± 12.5	19.5 ± 11.6	15.5 ± 12.6	0.191	0,666
**ICH**	In lifetime	46 (64.8%)	8 (80.0%)	38 (62.3%)	0.277	0,675
**FND**	In lifetime	34 (47.9%)	5 (50.0%)	29 (47.5%)	0.885	0,933
**Seizures**	In lifetime	30 (42.3%)	8 (80.0%)	22 (36.1%)	**0.009**	0,117
**Headache**	In lifetime	54 (76.1%)	5 (50.0%)	49 (80.3%)	**0.037**	0,361
**Cutaneous angiomas**	Baseline	25 (35.7%)	3 (30.0%)	22 (36.7%)	0.684	0,861
**Ocular angiomas**	Baseline	2 (2.9%)	0	2 (3.3%)	0.558	0,836
**Orbital angiomas**	Baseline	2 (2.9%)	1 (10.0%)	1 (1.7%)	0.143	0,666
**Vertebral vascular lesions**	Baseline	3 (8.6%)	1 (14.3%)	2 (7.1%)	0.546	0,836
**Spinal angioma**	Baseline	5 (13.5%)	2 (28.6%)	3 (10.0%)	0.196	0,666
**Total Cholesterol** (mg/dL)	baseline	178 [160-202]	172 [167-212]	180 [159-201]	0.667	0,861
**HDL Cholesterol** (mg/dL)	baseline	53 [43-62]	54 [50-72]	53 [42-62]	0.173	0,666
**Triglycerides** (mg/dL)	baseline	88 [73-136]	85 [65-129]	88 [73-139]	0.722	0,880
**CRP** (2mg/L)	baseline	1.07 [0.42-1.83]	0.93 [0.46-1.70]	1.07 [0.42-2.04]	0.934	0,959
**Vitamin D** (ng/mL**)**	baseline	18.0 [12.4-25.0]	15.9 [11.8-25.2]	19.0 [12.6-25.4]	0.541	0,836
**Creatinine** (mg/L)	baseline	0.73 [0.66-0.85]	0.78 [0.65-0.92]	0.73 [0.65-0.81]	0.554	0,836
**Sodium** (mEq/L)	baseline	141 [140-143]	142 [140-143]	141 [140-143]	0.453	0,836
**Potassium** (mEq/L)	baseline	4.3 [4.1-4.6]	4.4 [4.1-4.8]	4.3 [4.1-4.6]	0.300	0,688
**Glycaemia** (mg/L)	baseline	90.5 [85.3-96.8]	87 [84-93]	92 [86-99]	0.092	0,666
**ALT** (U/L)	baseline	17.5 [14.0-25.0]	16 [12-21]	19 [14-26]	0.259	0,675
**AST** (U/L)	baseline	18.0 [15.0-22.0]	19 [18-22]	17 [15-22]	0.222	0,666
**Modified Rankin Scale score**	Baseline	1.0 [0-1.0]	1 [0.75-1.0]	1 [0-1.5]	0.778	0,914
**Antiseizure treatment**	Baseline	33 (46.5%)	7 (70.0%)	26 (42.6%)	0.108	0,666
**Antihypertensive treatment**	Baseline	16 (22.5%)	3 (30.0%)	13 (21.3%)	0.542	0,836
**Antidepressants treatment**	Baseline	9 (12.7%)	0	9 (14.8%)	0.194	0,666
**New symptomatic ICH**	during FU	2 (2.8%)	2 (20.0%)	0	**$4.0x10^{-4}$**	0,016
**New symptomatic FND**	during FU	3 (4.2%)	2 (20.0%)	1 (1.6%)	**0.007**	0,117
**Epileptic seizures**	during FU	3 (4.2%)	0	3 (4.9%)	0.474	0,836
**Development of ≥5 denovo lesions**	during FU	32 (45.1%)	4 (40.0%)	28 (45.9%)	0.830	0,925
**Development of ≥10 denovo lesions**	during FU	17 (23.9%)	3 (30.0%)	14 (23.0%)	0.555	0,836

Data is represented as mean ± standard deviation, median [Q1-Q3] or N (%);p for Kruskal-Wallis or Chi2 test. BMI=body mass index; BP=blood pressure; SBP=Systolic BP; DBP=Diastolic BP; HR=heart rate; ICH: intracerebral haemorrhage; FND=focal neurological deficit; HDL=High-density lipoprotein; CRP=C reactive protein; ALT=Alanine Aminotransferase; AST=Aspartate Aminotransferase.

Supplementary Table 2 – Baseline characteristics of patients with FCCM who were depressed at baseline**.**

		**Not depressed (N=43)** (score of <=9 on BDI)	**depressed (N=20)** (score of =>10 on BDI)	**P**	**P** $_{fdr}$
**Age** (years)	Baseline	42.7 ± 13.8	53.0 ± 15.0	**0.010**	**0,049**
**Sex** (female)	Baseline	22 (51.2%)	13 (65.0%)	0.304	0,587
**BMI** (kg/m^2)	Baseline	23.9 ± 3.3	25.1 ± 4.0	0.236	0,587
**Randomized to propranolol**	Baseline	30 (69.8%)	13 (65.0%)	0.705	0,828
**Mean dose**	Baseline	70.8 ± 39.9	81.7 ± 54.0	0.766	0,828
**Systolic BP** (mmHg)	Baseline	121.2 ± 15.0	124.7 ± 14.2	0.357	0,587
**Diastolic BP** (mmHg)	Baseline	80.0 ± 8.3	76.8 ± 8.9	0.312	0,587
**Heart rate** (bpm)	Baseline	71.2 ± 12.6	73.8 ± 11.6	0.228	0,587
**Genetics**	KRIT1	31 (72.1%)	16 (80.0%)	0.766	0,828
	MGC4607	8 (18.6%)	3 (15.0%)		
	PDCD10	4 (9.3%)	1 (5.0%)		
**Time since diagnosis**	Baseline	14.1 ± 11.9	20.2 ± 13.5	0.055	0,202
**ICH**	In lifetime	25 (58.1%)	14 (70.0%)	0.367	0,587
**FND**	In lifetime	17 (39.5%)	13 (65.0%)	0.060	0,203
**Epileptic seizures**	In lifetime	16 (37.2%)	8 (40.0%)	0.832	0,851
**Headache**	In lifetime	33 (76.7%)	16 (80.0%)	0.772	0,828
**Cutaneous angiomas**	Baseline	15 (35.7%)	8 (40.0%)	0.744	0,828
**Ocular angiomas**	Baseline	2 (4.8%)	0	0.321	0,587
**Orbital angiomas**	Baseline	1 (2.4%)	0	0.487	0,670
**Vertebral angioma**	Baseline	2 (9.5%)	0	0.338	0,587
**Spinal angioma**	Baseline	2 (9.1%)	2 (20.0%)	0.387	0,587
**Total Cholesterol** (mg/dL)	Baseline	178 [158-199]	195 [163-215]	0.177	0,556
**HDL Cholesterol** (mg/dL)	Baseline	51.5 [42.1-61.7]	54.6 [41.8-65.1]	0.595	0,748
**Triglycerides** (mg/dL)	Baseline	84 [73-108]	125 [75-153]	0.030	0,132
**CRP** (mg/L)	Baseline	0.70 [0.31-1.30]	1.75 [0.76-4.44]	**0.005**	**0,028**
**Vitamin D** (ng/mL**)**	Baseline	19.0 [12.7-25.8]	17.0 [10.5-24.6]	0.376	0,587
**Creatinine** (mg/L)	Baseline	0.73 [0.63-0.88]	0.74 [0.70-0.85]	0.421	0,603
**Sodium** (mEq/L)	Baseline	141 [140-143]	142 [140-143]	0.556	0,741
**Potassium** (mEq/L)	Baseline	4.3 [4.1-4.6]	4.3 [4.0-4.4]	0.275	0,587
**Glycaemia** (mg/L)	Baseline	93.0 [86.5-97.0]	90.0 [86.0-101.0]	0.959	0,959
**ALT** (U/L)	Baseline	18 [14-24]	17 [14-32]	0.425	0,603
**AST** (U/L)	Baseline	17 [15-23]	18 [15-20]	0.811	0,850
**Modified Rankin scale score**	Baseline	0 [0-1]	1 [1-3]	**0.003**	**0,019**
**Antiseizure treatment**	Baseline	17 (39.5%)	11 (55.0%)	0.250	0,587
**Antihypertensive treatment**	Baseline	7 (16.3%)	8 (40.0%)	0.040	0,160
**Antidepressants treatment**	Baseline	2 (4.7%)	7 (35.0%)	**0.001**	**0,007**
**New symptomatic ICH**	during FU	1 (2.3%)	1 (5.0%)	0.573	0,742
**New symptomatic FND**	during FU	2 (4.7%)	0	0.327	0,587
**Epileptic Seizures**	during FU	3 (7.0%)	0	0.226	0,587
**Development of ≥5 denovo lesions**	during FU	17 (42.5%)	11 (57.9%)	0.269	0,587
**Development of ≥10 denovo lesions**	during FU	10 (25.0%)	4 (21.1%)	0.739	0,828
**BDI score**	Baseline	5 [3-6]	14 [11-20]	**$1.9x10^{-10}$**	**$8.8x10^{-9}$**
**STAI X-1 score**	Baseline	36 [30-43]	48 [42-58]	**$2.1x10^{-5}$**	**$3.1x10^{-4}$**
**STAI X-2 score**	Baseline	35 [30-41]	52 [45-57]	**$3.3x10^{-8}$**	**$7.3x10^{-7}$**
**SF36 PCS**	Baseline	512 [351-631]	300 [148-412]	**0.001**	**0,007**
**SF36 MCS**	Baseline	482 [333-574]	315 [203-378]	**$4.1x10^{-4}$**	**0,005**

Data is represented as mean ± standard deviation, median [Q1-Q3] or N (%); p for Kruskal-Wallis or Chi2 test. BMI=body mass index; BP=blood pressure; SBP=Systolic BP; DBP=Diastolic BP; HR=heart rate; ICH: intracerebral haemorrhage; FND=focal neurological deficit; HDL=High-density lipoprotein; CRP=C reactive protein; ALT=Alanine Aminotransferase; AST=Aspartate Aminotransferase.

Supplementary Table 3 – Baseline characteristics of patients with FCCM who were anxious at baseline**.**

		Not anxious (N=65)	anxious (N=6)	P	P_{fdr}
**Age** (years)	Baseline	44.2 ± 13.9	60.2 ± 18.5	**0.013**	0,082
**Sex** (female)	Baseline	36 (55.4%)	4 (66.7%)	0.594	0,738
**BMI** (kg/m^2)	Baseline	24.1 ± 3.6	23.6 ± 3.6	0.393	0,721
**Randomized to propranolol**	Baseline	45 (69.2%)	3 (50.0%)	0.335	0,673
**Mean dose**	Baseline	75.5 ± 45.1	45.0 ± 5.0	0.411	0,723
**Systolic BP** (mmHg)	Baseline	121 ± 14	127 ± 13	0.345	0,673
**Diastolic BP** (mmHg)	Baseline	79.1 ± 8.2	73.5 ± 8.1	0.187	0,502
**Heart rate** (bpm)	Baseline	71.2 ± 8.2	74.0 ± 18.7	0.928	0,950
**Genetics**	KRIT1	51 (78.559	3 (50.0%)	0.287	0,665
	MGC4607	10 (15.4%)	2 (33.3%)		
	PDCD10	4 (6.2%)	1 (16.7%)		
**Time since diagnosis**	Baseline	15.4 ± 11.6	23.0 ± 19.6	0.325	0,673
**ICH**	In lifetime	42 (64.6%)	4 (66.7%)	0.920	0,950
**FND**	In lifetime	31 (47.7%)	3 (50.0%)	0.914	0,950
**Epileptic seizures**	In lifetime	28 (43.1%)	2 (33.3%)	0.644	0,745
**Headache**	In lifetime	50 (76.9%)	4 (66.7%)	0.573	0,738
**Cutaneous angiomas**	Baseline	22 (34.4%)	3 (50.0%)	0.445	0,738
**Ocular angiomas**	Baseline	2 (3.1%)	0	0.660	0,745
**Orbital angiomas**	Baseline	2 (3.1%)	0	0.660	0,745
**Vertebral angioma**	Baseline	3 (9.7%)	0	0.515	0,738
**Spinal angioma**	Baseline	3 (9.1%)	2 (50.0%)	**0.024**	0,117
**Total Cholesterol** (mg/dL)	Baseline	180 [160-202]	175 [157-214]	0.951	0,951
**HDL Cholesterol** (mg/dL)	Baseline	51.8 [42.4-61.8]	56.8 [51.8-69.7]	0.207	0,506
**Triglycerides** (mg/dL)	Baseline	86 [73-128]	143 [71-147]	0.352	0,673
**CRP** (mg/L)	Baseline	0.98 [0.42-1.81]	1.57 [1.04-4.10]	0.193	0,502
**Vitamin D** (ng/mL**)**	Baseline	18.0 [12.6-25.0]	21.9 [9.8-25.9]	0.901	0,950
**Creatinine** (mg/L)	Baseline	0.73 [0.64-0.85]	0.77 [0.70-1.03]	0.173	0,502
**Sodium** (mEq/L)	Baseline	141 [140-142]	143 [142-144]	**0.022**	0,117
**Potassium** (mEq/L)	Baseline	4.3 [4.1-4.6]	4.4 [4.2-5.0]	0.477	0,738
**Glycaemia** (mg/L)	Baseline	91 [86-97]	88 [85-96]	0.604	0,738
**ALT** (U/L)	Baseline	17 [14-25]	18 [14-41]	0.523	0,738
**AST** (U/L)	Baseline	17 [15-22]	20 [19-23]	0.142	0,502
**Modified Rankin scale score**	Baseline	1.0 [0-1.0]	1.5 [0.8-3.0]	0.194	0,502
**Antiseizure treatment**	Baseline	32 (49.1%)	1 (16.7%)	0.126	0,502
**Antihypertensive treatment**	Baseline	14 (21.5%)	2 (33.3%)	0.508	0,738
**Antidepressants treatment**	Baseline	6 (9.2%)	3 (50.0%)	0.004	**0,029**
**New symptomatic ICH**	during FU	1 (1.5%)	1 (16.7%)	0.032	0,141
**New symptomatic FND**	during FU	3 (4.6%)	0	0.591	0,738
**Epileptic seizures**	during FU	3 (4.6%)	0	0.591	0,738
**Development of ≥5 denovo lesions**	during FU	29 (46.8%)	3 (60.0%)	0.569	0,738
**Development of ≥10 denovo lesions**	during FU	17 (27.4%)	0	0.175	0,502
**BDI score**	Baseline	5.5 [3.8-10.0]	24.0 [12.0-25.5]	**3.2×10^{-4}**	**0,004**
**STAI X-1 score**	Baseline	40 [31-46]	58 [54-65]	**8.5×10^{-5}**	**0,001**
**STAI X-2 score**	Baseline	39 [33-44]	60 [55-69]	**1.1×10^{-5}**	**4.8×10^{-4}**
**SF36 PCS**	Baseline	413 [311-593]	151 [133-258]	**0.001**	**0,009**
**SF36 MCS**	Baseline	429 [319-524]	143 [65-255]	**7.5×10^{-5}**	**0,001**

Data is represented as mean ± standard deviation, median [Q1-Q3] or N (%);p for Kruskal-Wallis or Chi2 test. BMI=body mass index; BP=blood pressure; SBP=Systolic BP; DBP=Diastolic BP; HR=heart rate; ICH: intracerebral haemorrhage; FND=focal neurological deficit; HDL=High-density lipoprotein; CRP=C reactive protein; ALT=Alanine Aminotransferase; AST=Aspartate Aminotransferase.

Supplementary Table 4 – Baseline characteristics of patients with FCCM with respect to Mental Component Scale (MCS) at baseline**.**

		MCS <50 N=33	MCS => 50 N=38	P-value*	P_{fdr}
**Age** (years)	Baseline	48.5 ± 16.5	42.9 ± 13.0	0.089	0,402
**Sex** (female)	Baseline	18 (54.5%)	22 (57.9%)	0.777	0,855
**BMI** (kg/m^2)	Baseline	24.6 ± 3.9	23.5 ± 3.3	0.253	0,506
**Randomized to propranolol**	Baseline	24 (72.7%)	24 (63.2%)	0.390	0,646
**Mean dose**	Baseline	77.3 ± 47.5	70.1 ± 40.6	0.629	0,762
**Systolic BP** (mmHg)	Baseline	124 ± 14	120 ± 14	0.329	0,581
**Diastolic BP** (mmHg)	Baseline	78 ± 8	79 ± 9	0.679	0,782
**Heart rate** (bpm)	Baseline	72 ± 11	71 ± 13	0.415	0,646
**Genetics**	KRIT1	24 (72.7%)	30 (78.9%)	0.292	0,559
	MGC4607	5 (15.2%)	7 (18.4%)		
	PDCD10	4 (12.1%)	1 (2.6%)		
**Time since diagnosis**	Baseline	17.6 ± 13.5	14.7 ± 11.5	0.330	0,581
**ICH**	In lifetime	24 (72.7%)	22 (57.9%)	0.192	0,484
**FND**	In lifetime	19 (57.6%)	15 (39.5%)	0.128	0,402
**Epileptic seizures**	In lifetime	15 (45.5%)	15 (39.5%)	0.611	0,762
**Headache**	In lifetime	23 (69.7%)	31 (81.6%)	0.242	0,506
**Cutaneous angiomas**	Baseline	14 (43.8%)	11 (28.9%)	0.198	0,484
**Ocular angiomas**	Baseline	0	2 (5.3%)	0.188	0,484
**Orbital angiomas**	Baseline	0	2 (5.3%)	0.188	0,484
**Vertebral angioma**	Baseline	1 (5.3%)	2 (12.5%)	0.446	0,646
**Spinal angioma**	Baseline	4 (20.0%)	1 (5.9%)	0.211	0,489
**Total Cholesterol** (mg/dL)	Baseline	180 [163-205]	176 [158-200]	0.397	0,646
**HDL Cholesterol** (mg/dL)	Baseline	53.4 [43.5-63.9]	52.4 [42.8-62.5]	0.876	0,896
**Triglycerides** (mg/dL)	Baseline	86 [71-143]	91 [74-135]	0.991	0,991
**CRP** (mg/L)	Baseline	1.45 [0.52-3.24]	0.73 [0.40-1.34]	**0.041**	0,248
**Vitamin D** (ng/mL**)**	Baseline	17.4 [11.7-24.3]	20.8 [12.9-26.5]	0.235	0,506
**Creatinine** (mg/L)	Baseline	0.75 [0.70-0.89]	0.70 [0.61-0.79]	**0.045**	0,248
**Sodium** (mEq/L)	Baseline	141 [140-143]	141 [140-142]	0.856	0,896
**Potassium** (mEq/L)	Baseline	4.3 [4.0-4.5]	4.4 [4.2-4.6]	0.118	0,402
**Glycaemia** (mg/L)	Baseline	90 [85-94]	93 [86-100]	0.455	0,646
**ALT** (U/L)	Baseline	17 [14-25]	18 [13-25]	0.614	0,762
**AST** (U/L)	Baseline	18.0 [15.8-20.8]	17.0 [14.0-22.5]	0.624	0,762
**Modified Rankin scale score**	Baseline	1 [0-2]	1 [0-1]	0.122	0,402
**Antiseizure treatment**	Baseline	17 (51.5%)	16 (42.1%)	0.428	0,646
**Antihypertensive treatment**	Baseline	11 (33.3%)	5 (13.2%)	**0.042**	0,248
**Antidepressants treatment**	Baseline	7 (21.2%)	2 (5.3%)	**0.044**	0,248
**New symptomatic ICH**	during FU	2 (6.1%)	0	0.124	0.402
**New symptomatic FND**	during FU	1 (3.0%)	2 (5.3%)	0.641	0,762
**Epileptic seizures**	during FU	1 (3.0%)	2 (5.3%)	0.641	0,762
**Development of ≥5 denovo lesions**	during FU	14 (45.2%)	18 (50.0%)	0.693	0,782
**Development of ≥10 denovo lesions**	during FU	5 (16.1%)	12 (33.3%)	0.107	0,402
**BDI score**	Baseline	10.0 [6.5-17.0]	4.5 [3.0-6.0]	**$8.0x10^{-6}$**	**$2,2x10^{-4}$**
**STAI X-1 score**	Baseline	45.5 [39.8-56.0]	35.0 [30.0-41.5]	**$3.6x10^{-4}$**	**0,004**
**STAI X-2 score**	Baseline	44.5 [36.8-54.0]	33.5 [29.0-41.5]	**$5.3x10^{-5}$**	**0,001**
**SF36 PCS**	Baseline	46.1 [38.1-51.5]	48.8 [36.6-52.1]	0.872	0,896
**SF36 MCS**	Baseline	31.9 [24.4-41.3]	60.9 [58.8-63.0]	**$4.9x10^{-13}$**	**$2,2x10^{-11}$**

Data is represented as mean ± standard deviation, median [Q1-Q3] or N (%);p for Kruskal-Wallis or Chi2 test. BMI=body mass index; BP=blood pressure; SBP=Systolic BP; DBP=Diastolic BP; HR=heart rate; ICH: intracerebral haemorrhage; FND=focal neurological deficit; HDL=High-density lipoprotein; CRP=C reactive protein; ALT=Alanine Aminotransferase; AST=Aspartate Aminotransferase.

Supplementary Table 5 – Baseline characteristics of patients with FCCM with respect to Physical Component Scale (PCS) at baseline**.**

		PCS <50<br>N=43	PCS => 50<br>N=28	P	P_{fdr}
**Age** (years)	Baseline	49.2 ± 15.2	39.9 ± 12.7	**0.020**	0,098
**Sex** (female)	Baseline	26 (60.5%)	14 (50.0%)	0.385	0,717
**BMI** (kg/m^2)	Baseline	24.1 ± 3.7	23.8 ± 3.6	0.723	0,816
**Randomized to propranolol**	Baseline	30 (69.8%)	18 (64.3%)	0.630	0,816
**Mean dose**	Baseline	78.3 ± 49.3	66.3 ± 34.8	0.657	0,816
**Systolic BP** (mmHg)	Baseline	123 ± 15	120 ± 13.1	0.722	0,816
**Diastolic BP** (mmHg)	Baseline	78 ± 8	79 ± 9	0.514	0,808
**Heart rate** (bpm)	Baseline	73 ± 13	69 ± 10	0.183	0,447
**Genetics**	KRIT1	30 (69.8%)	24 (85.7%)	0.205	0,475
	MGC4607	10 (23.3%)	2 (7.1%)		
	PDCD10	3 (7.0%)	2 (7.1%)		
**Time since diagnosis**	Baseline	18.6±13.8	12.2 ± 9.1	0.056	0.176
**ICH**	In lifetime	30 (69.8%)	16 (57.1%)	0.276	0.576
**FND**	In lifetime	27 (62.8%)	7 (25.0%)	**0.002**	**0,015**
**Epileptic seizures**	In lifetime	18 (41.9%)	12 (42.9%)	0.934	0,956
**Headache**	In lifetime	34 (79.1%)	20 (71.4%)	0.461	0,759
**Cutaneous angiomas**	Baseline	14 (33.3%)	11 (39.3%)	0.611	0,816
**Ocular angiomas**	Baseline	0	2 (7.1%)	0.079	0,232
**Orbital angiomas**	Baseline	1 (2.4%)	1 (3.6%)	0.770	0,847
**Vertebral angioma**	Baseline	2 (10.5%)	1 (6.3%)	0.653	0,816
**Spinal angioma**	Baseline	3 (14.3%)	2 (12.5%)	0.875	0,917
**Total Cholesterol** (mg/dL)	Baseline	190 [166-205]	172 [158-189]	**0.044**	0,161
**HDL Cholesterol** (mg/dL)	Baseline	53.7 [42.6-66.4]	52.3 [44.0-60.0]	0.466	0,759
**Triglycerides** (mg/dL)	Baseline	97 [78-144]	78 [57-107]	**0.015**	0,083
**CRP** (mg/L)	Baseline	1.25 [0.52-2.69]	0.67 [0.34-1.41]	**0.040**	0,160
**Vitamin D** (ng/mL**)**	Baseline	20.1 [13.0-25.7]	17.7 [11.9-24.7]	0.707	0,816
**Creatinine** (mg/L)	Baseline	0.73 [0.68-0.87]	0.73 [0.60-0.82]	0.391	0,717
**Sodium** (mEq/L)	Baseline	141 [140-142]	141 [140-143]	0.632	0,816
**Potassium** (mEq/L)	Baseline	4.3 [4.1-4.5]	4.4 [4.2-4.7]	0.288	0,576
**Glycaemia** (mg/L)	Baseline	93 [86-101]	88 [85-93]	0.121	0,313
**ALT** (U/L)	Baseline	16.5 [14.0-25.0]	19.5 [14.0-26.3]	0.627	0,816
**AST** (U/L)	Baseline	18 [16-22]	17 [14-24]	0.439	0,759
**Modified Rankin scale score**	Baseline	1 [1-3]	0 [0-1]	**$7.1x10^{-7}$**	**$1,5x10^{-5}$**
**Antiseizure treatment**	Baseline	20 (46.5%)	13 (46.3%)	0.995	0,995
**Antihypertensive treatment**	Baseline	13 (30.2%)	3 (10.7%)	0.054	0,176
**Antidepressants treatment**	Baseline	9 (20.9%)	0	**0.010**	0,063
**New symptomatic ICH**	during FU	2 (4.7%)	0	0.247	0,543
**New symptomatic FND**	during FU	2 (4.7%)	1 (3.6%)	0.825	0,885
**Epileptic seizures**	during FU	0	3 (10.7%)	**0.028**	0,123
**Development of ≥5 denovo lesions**	during FU	21 (50.0%)	11 (44.0%)	0.634	0,816
**Development of ≥10 denovo lesions**	during FU	10 (23.8%)	7 (28.0%)	0.703	0,816
**BDI score**	Baseline	9.0 [5.0-15.5]	4.0 [2.0-6.5]	**$3.9x10^{-4}$**	**0,004**
**STAI X-1 score**	Baseline	44.5 [38.5-52.3]	36.0 [30.0-40.5]	**0.001**	**0,009**
**STAI X-2 score**	Baseline	43.5 [37.0-52.0]	33.0 [28.8-42.3]	**$3.4x10^{-4}$**	**0,004**
**SF36 PCS**	Baseline	42.0 [34.6-45.5]	52.4 [49.8-54.6]	**$1.4x10^{-12}$**	**$6,2x10^{-11}$**
**SF36 MCS**	Baseline	44.2 [28.5-61.0]	58.6 [43.4-61.0]	0.102	0,281

Data is represented as mean ± standard deviation, median [Q1-Q3] or N (%); p for Kruskal-Wallis or Chi2 test. BMI=body mass index; BP=blood pressure; SBP=Systolic BP; DBP=Diastolic BP; HR=heart rate; ICH: intracerebral haemorrhage; FND=focal neurological deficit; HDL=High-density lipoprotein; CRP=C reactive protein; ALT=Alanine Aminotransferase; AST=Aspartate Aminotransferase.

Supplementary Figure 1 – SF-36 domains for Treat_CCM groups and the Italian general population

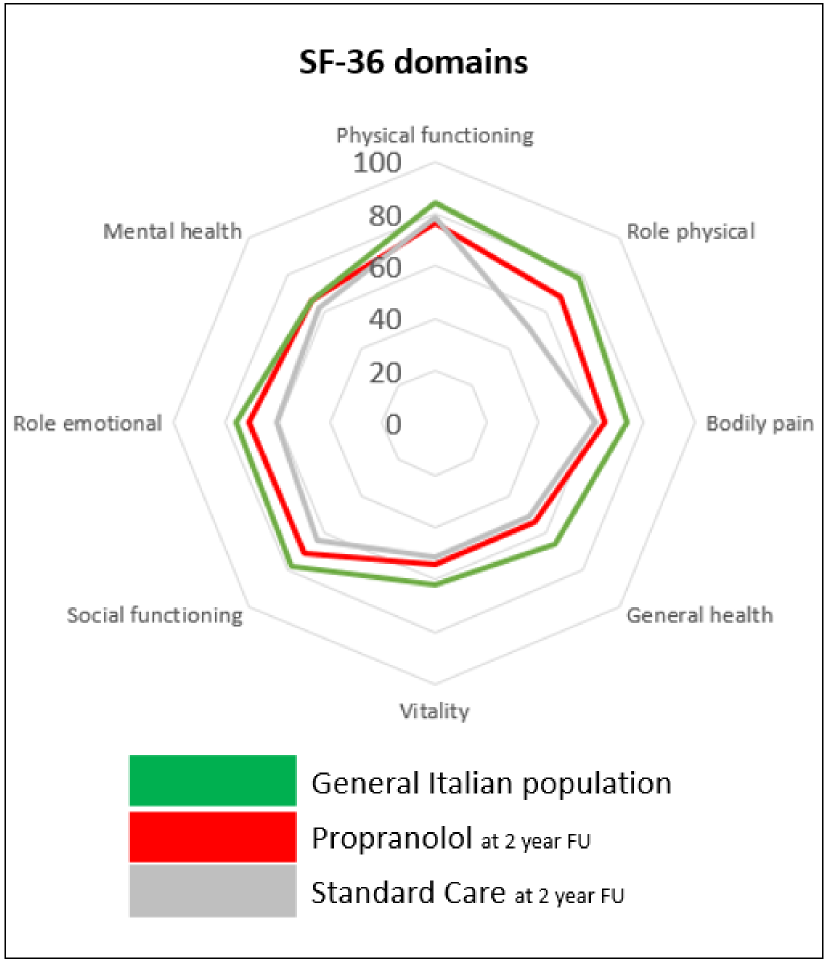

	Italian population	Propranolol at 2 year FU	Standard care at 2 year FU	P propranolol vs standard care
Physical functioning	84,5 ± 23,2	76,7 ± 24,2	78,4 ± 30,3	0,385
Role physical	78,2 ± 35,9	68,1 ± 39,9	51,1 ± 43,3	0,087
Bodily pain	73,7 ± 27,7	65,2 ± 29,3	61,8 ± 27,3	0,563
General health	65,2 ± 22,2	54,0 ± 23,5	51,1 ± 15,1	0,536
Vitality	61,9 ± 20,7	54,3 ± 19,6	51,1 ± 17,8	0,497
Social functioning	77,4 ± 23,3	70,3 ± 23,0	63,9 ± 23,9	0,311
Role emotional	76,2 ± 37,3	70,9 ± 42,1	60,5 ± 42,0	0,262
Mental health	66,6 ± 20,9	66,5 ± 19,8	62,2 ± 19,4	0,213

	Italian population	Propranolol at 2 year FU	Standard care at 2 year FU	P propranolol vs standard care
PCS	52,7 ± 7,7	45,1 ± 9,3	44,7 ±10,5	0,990
MCS	47,6 ± 10,1	50,2 ± 15,1	46,0 ± 15,4	0,198
